# Comparison of effectiveness of common targeting heuristics in repetitive transcranial magnetic stimulation treatment of depression

**DOI:** 10.1136/bmjment-2025-301598

**Published:** 2025-05-14

**Authors:** Katrin Sakreida, Nicholas T Trapp, Sarah Kreuzer, Ulrike Rubin, Dieter Schnabel, Jana Hovančáková, Alexander T Sack, Irene Neuner, Thomas Frodl, Timm B Poeppl

**Affiliations:** 1 Department of Psychiatry, Psychotherapy and Psychosomatics, RWTH Aachen University, Aachen, Germany; 2 Department of Psychiatry, University of Iowa, Iowa City, Iowa, USA; 3 Iowa Neuroscience Institute, University of Iowa, Iowa City, Iowa, USA; 4 Department of Psychiatry and Psychotherapy, University of Regensburg, Regensburg, Germany; 5 Department of General Psychiatry, LVR-Klinik Düren, Düren, Germany; 6 Department of Cognitive Neuroscience, Maastricht University, Maastricht, The Netherlands

**Keywords:** Cross-Sectional Studies, Data Interpretation, Statistical, Depression, PSYCHIATRY

## Abstract

**Background:**

Repetitive transcranial magnetic stimulation (rTMS) of the left dorsolateral prefrontal cortex (DLPFC) is an effective non-pharmacological, non-invasive intervention for depression. However, the optimal strategy for localising the DLPFC treatment site on the patient’s scalp is heavily disputed. Routine strategies were previously incrementally refined and compared in terms of anatomical accuracy, but little is known about their impact on clinical outcomes.

**Objective:**

To assess the impact of three common scalp-based heuristics for magnetic coil positioning on the treatment outcome of rTMS.

**Methods:**

This retrospective analysis of real-world clinical data involved patients suffering from a major depressive episode (n=94) who received a 4-week course of excitatory rTMS to the left DLPFC. The treatment target (ie, coil position) was either determined at an absolute distance anterior to the motor hotspot (‘6 cm rule’) or defined in reference to the EEG electrode position F3 using a traditional (‘Beam F3’) or optimised (‘Beam F3 Adjusted’) approach.

**Findings:**

There was no statistically significant difference between the ‘6 cm rule’ and the ‘Beam F3’ method nor between the ‘Beam F3’ and the ‘Beam F3 Adjusted’ method in head-to-head comparisons of averaged per cent change of scores on depression rating scales (all p>0.605) and response rate (all p>0.475).

**Conclusions:**

Enhancing targeting precision via scalp-based heuristics does not affect treatment outcomes.

**Clinical implications:**

There is no need for clinicians to switch from their familiar to an ‘advanced’ approach among these common targeting heuristics.

## Introduction

High-frequency repetitive transcranial magnetic stimulation (rTMS) of the left dorsolateral prefrontal cortex (DLPFC) is an effective intervention for the treatment of depression.^1^ However, where to place the TMS coil on the patient’s scalp in order to target the optimal location within the DLPFC is heavily disputed. In clinical practice, most TMS coil positioning approaches use scalp-based heuristics for localising the DLPFC depression target. These include the ‘5 cm rule’, ‘5.5 cm rule’ and ‘6 cm rule’, determining the treatment target at an absolute distance anterior to the cortical hand ‘motor hotspot’. Also, the ‘Beam F3’ and ‘adjusted Beam F3’ methods are commonly applied, defining the target in reference to the EEG electrode position F3.^2–4^ While these methods were incrementally refined and compared in terms of anatomical accuracy,^5^ little is known about their impact on clinical outcomes. A recent head-to-head comparison between the ‘5.5 cm’ and the ‘Beam F3’ targeting methods failed to find clinical efficacy differences in rTMS treatment of depression.^6^ Here, we use real-world clinical data to retrospectively assess whether the ‘Beam F3’ method is superior to the ‘6 cm rule’ and if the ‘adjusted Beam F3’ is an enhancement of the original ‘Beam F3’ method in terms of clinical outcome.

## Methods

Sample 1 comprised 46 patients who were treated at RWTH Aachen University Hospital. Sample 2 comprised 48 patients who were treated at LVR-Klinik Düren (sample size estimations and characteristics in online supplemental file 1). Eligibility for inclusion in the analysis required a primary psychiatric diagnosis of moderate or severe major or bipolar depression according to ICD-10. Patients remained on prescribed medications; adjustments were made as clinically indicated throughout the treatment course.^6^ Excitatory rTMS was applied over the left DLPFC in 20 sessions/4 weeks (online supplemental file 1). Patients were blind to the localisation method (sample 1: ‘Beam F3’ n=23, ‘Beam F3 Adjusted’ n=23; sample 2: ‘Beam F3’ n=24, ‘6 cm rule’ n=24).

10.1136/bmjment-2025-301598.supp1 Supplementary data

Depression severity was assessed using the self-report Beck Depression Inventory revised (BDI–II) as well as the clinician-administered Hamilton Depression Rating Scale (HAMD-17) in sample 1 and the Beck Depression Inventory (BDI) in sample 2. Treatment primary outcome was operationalised by calculating the per cent change of depression scores from baseline to end of intervention. ‘Response’ was defined as a sum score reduction of ≥50% from baseline to the end of treatment (statistical procedures in online supplemental file 1).

## Results

**Figure 1:**
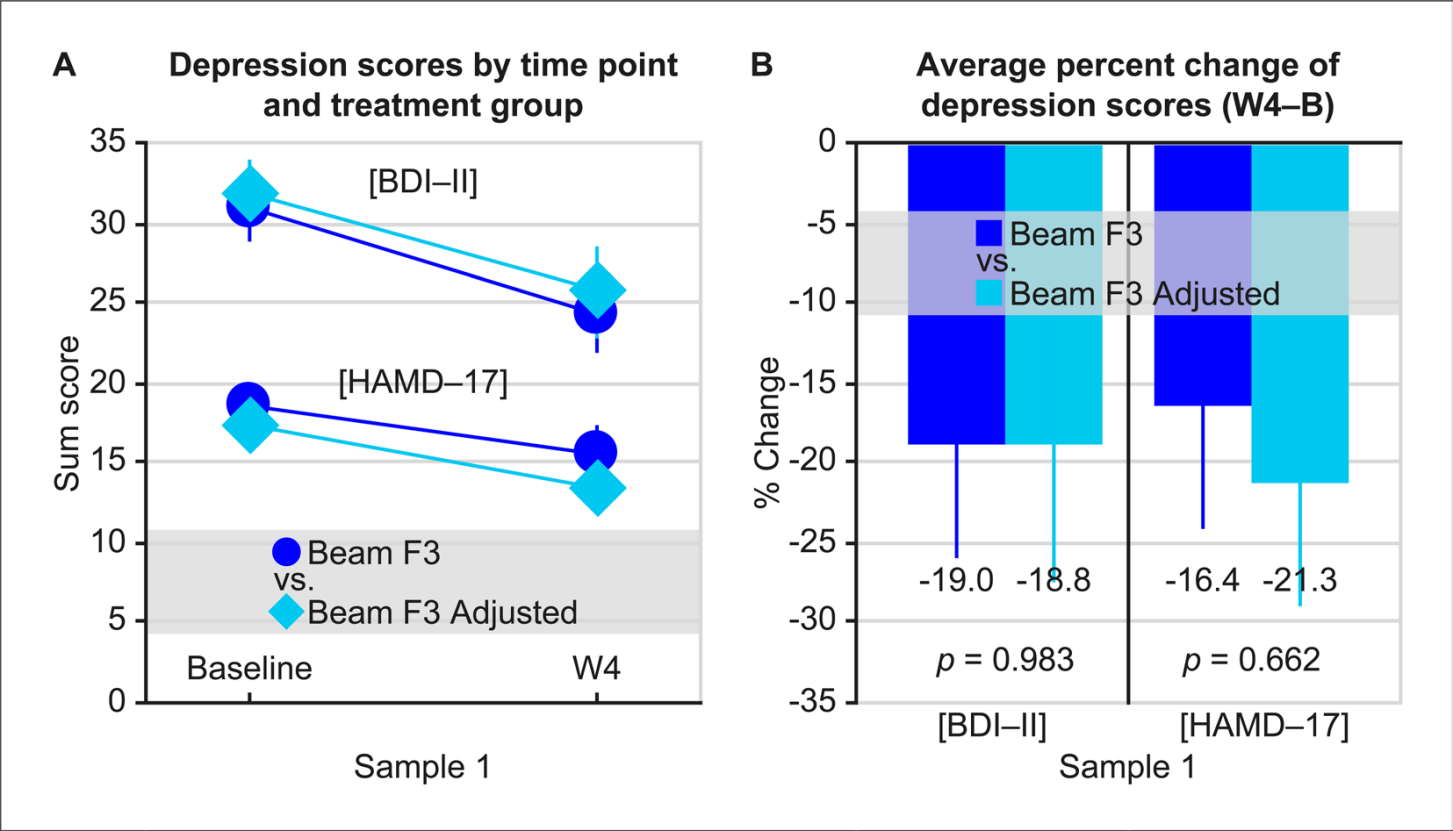
Treatment outcomes of localisation methods ‘Beam F3’ vs ‘Beam F3 Adjusted’ in sample 1 (Aachen). There was neither an interaction between ‘localisation method’ and ‘timepoint’ on depression scores (A) nor a difference in change of depression severity between the localisation methods (B). The main effect was highly significant for the factor ‘timepoint’ (BDI–II: F_1,44_=14.151, p<0.001, η^2^_p_=0.243; HAMD-17: F_1,44_=14.726, p<0.001, η^2^_p_=0.251), indicating that depression severity improved during treatment. Mean and SE of the mean are displayed. B, Baseline; BDI–II, Beck Depression Inventory revised; HAMD-17, Hamilton Depression Rating Scale; W4, after week 4/20 sessions of treatment.

There was no difference between ‘Beam F3’ and ‘Beam F3 Adjusted’ in head-to-head comparisons of averaged per cent change of depression scores in sample 1 (BDI–II: −19.0±7.0 vs −18.8±8.8 (M±SEM), t_44_=−0.021, p=0.983, d_z_=−0.006; HAMD-17: −16.4±7.7 vs −21.3±7.9 (M±SEM), t_44_=−0.440, p=0.662, d_z_=0.130; figure 1). The 2×2 ANOVA indicated no interaction between ‘localisation method’ and ‘timepoint’ (BDI–II: F_1,44_=0.016, p=0.901, η^2^_p_=0.000; HAMD-17: F_1,44_=0.205, p=0.653, η^2^_p_=0.005). Response rates were equally distributed (to non-response rates) across the ‘Beam F3’ and ‘Beam F3 Adjusted’ localisation methods (BDI–II: 26.1 vs 17.4 (%), χ^2^_1_=0.511, p=0.475, φ=0.105; HAMD-17: 13.0 vs 13.0 (%), χ^2^_1_=0.000, p=1.000, φ=0.000) (further details in online supplemental file 1).

**Figure 2:**
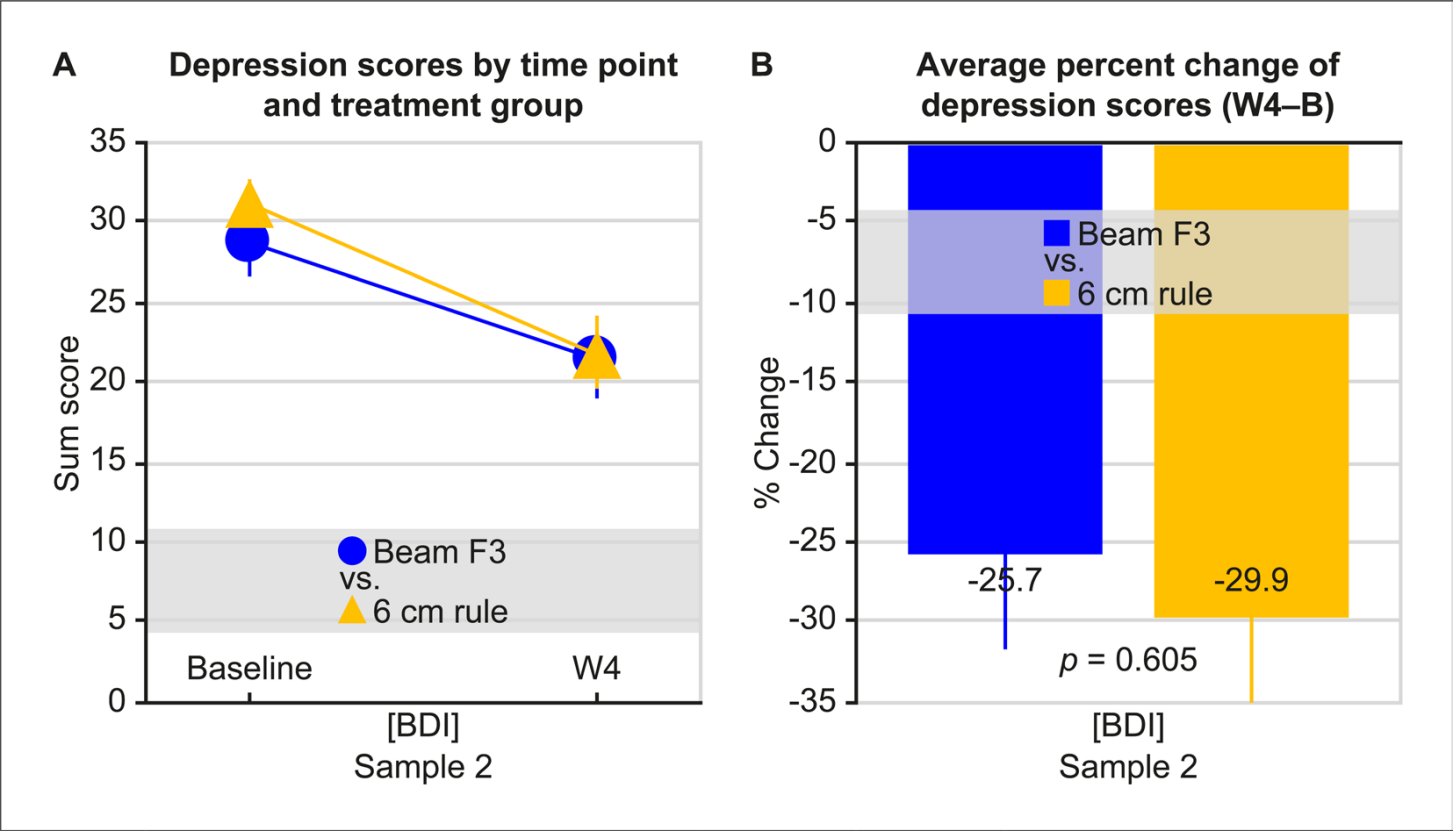
Treatment outcomes of localisation methods ‘Beam F3’ vs ‘6 cm rule’ in sample 2 (Düren). There was neither an interaction between ‘localisation method’ and ‘timepoint’ on depression scores (A) nor a difference in change of depression severity between the localisation methods (B). There was a highly significant main effect for the factor ‘timepoint’, indicating that depression severity was reduced during treatment (F_1,46_=43.021, p<0.001, η^2^_p_=0.483). Mean and SE of the mean are displayed. B, Baseline; BDI, Beck Depression Inventory; W4, after week 4/20 sessions of treatment.

There was also no difference between ‘Beam F3’ and the ‘6 cm rule’ in head-to-head comparisons of averaged per cent change of depression scores in sample 2 (BDI: −25.7±6.0 vs −29.9±5.1 (M±SEM), t_46_=0.520, p=0.605, d_z_=0.150; figure 2). Also here, the 2×2 ANOVA indicated no interaction between ‘localisation method’ and ‘timepoint’ (F_1,46_=0.579, p=0.450, η^2^_p_=0.012). Also across the ‘Beam F3’ and ‘6 cm rule’ localisation methods, response rates were equally distributed (BDI: 25.0 vs 25.0 (%), χ^2^_1_=0.000, p=1.000, φ=0.000).

## Discussion

A previous randomised trial demonstrated similar effectiveness of ‘Beam F3’ and ‘5.5 cm rule’ targeting in rTMS treatment of depression.^6^ Our retrospective analyses of real-world clinical data complement these findings by showing that the ‘Beam F3’ method is not superior to the ‘6 cm rule’ and that ‘adjusted Beam F3’ does not outmatch the original ‘Beam F3’ method in terms of clinical outcome. This study provides unique data showing similar depression symptom improvement among three common scalp-based heuristics for TMS coil positioning. Based on our results, the number of participants needed to achieve 80% power was determined to be between ≈1400 and ≈872 000 for the outcome of per cent change in depression scales (online supplemental file 1). Even if group differences could be detected with such enormous sample sizes, this would be unlikely to translate into clinically meaningful differences between targeting strategies. This relative robustness of clinical efficacy against various scalp-based targeting heuristics may be due to interindividual neuroanatomical variability in scalp-cortex correspondence. However, there is also no clear evidence that target localisation using individual, structural MRI-guided neuronavigation improves treatment outcome.^7^ In contrast, evidence is accumulating that individualised functional connectivity-guided precision may be crucial for the identification of the optimal rTMS treatment site for depression and boost treatment response.^8^ Our results indicate that enhancing targeting precision via scalp-based heuristics does not affect treatment outcome, and thus, there is no need for clinicians to switch from their familiar to an ‘advanced’ approach among these common targeting heuristics.

## References

[1] Mutz J, Vipulananthan V, Carter B, et al. Comparative efficacy and acceptability of non-surgical brain stimulation for the acute treatment of major depressive episodes in adults: systematic review and network meta-analysis. BMJ 2019;364:l1079. 10.1136/bmj.l1079 30917990 PMC6435996

[2] Fitzgerald PB. Targeting repetitive transcranial magnetic stimulation in depression: do we really know what we are stimulating and how best to do it? Brain Stimul 2021;14:730–6. 10.1016/j.brs.2021.04.018 33940242

[3] Beam W, Borckardt JJ, Reeves ST, et al. An efficient and accurate new method for locating the F3 position for prefrontal TMS applications. Brain Stimul 2009;2:50–4. 10.1016/j.brs.2008.09.006 20539835 PMC2882797

[4] Mir-Moghtadaei A, Caballero R, Fried P, et al. Concordance Between BeamF3 and MRI-neuronavigated Target Sites for Repetitive Transcranial Magnetic Stimulation of the Left Dorsolateral Prefrontal Cortex. Brain Stimul 2015;8:965–73. 10.1016/j.brs.2015.05.008 26115776 PMC4833442

[5] Trapp NT, Bruss J, King Johnson M, et al. Reliability of targeting methods in TMS for depression: Beam F3 vs. 5.5 cm. Brain Stimul 2020;13:578–81. 10.1016/j.brs.2020.01.010 32289680 PMC7507589

[6] Trapp NT, Pace BD, Neisewander B, et al. A randomized trial comparing beam F3 and 5.5 cm targeting in rTMS treatment of depression demonstrates similar effectiveness. Brain Stimul 2023;16:1392–400. 10.1016/j.brs.2023.09.006 37714408 PMC11095825

[7] Hebel T, Göllnitz A, Schoisswohl S, et al. A direct comparison of neuronavigated and non-neuronavigated intermittent theta burst stimulation in the treatment of depression. Brain Stimul 2021;14:335–43. 10.1016/j.brs.2021.01.013 33493624

[8] Cash RFH, Cocchi L, Lv J, et al. Functional Magnetic Resonance Imaging-Guided Personalization of Transcranial Magnetic Stimulation Treatment for Depression. JAMA Psychiatry 2021;78:337–9. 10.1001/jamapsychiatry.2020.3794 33237320 PMC7689561

