# Supplementary material for: Comparison of effectiveness of common targeting heuristics in repetitive transcranial magnetic stimulation treatment of depression
Source: BMJ Ment Health. 2025 May 14;28(1):e301598. doi: 10.1136/bmjment-2025-301598 (PMC13059834; doi:10.1136/bmjment-2025-301598)
Supplement: Supplementary data [file bmjment-28-1-s001.pdf]

Supplementary material

Supplementary Methods

Sample size estimation

We probed whether the employed sample sizes were appropriate for investigating the hypotheses (1) that “Beam F3 Adjusted” is superior to “Beam F3” and (2) that “Beam F3” is superior to the “6 cm rule”. The sample size estimation was aimed at detecting, between groups, the minimum clinically important difference (MCID)[1] in percentaged Beck Depression Inventory score change[2-7]. With an α of 0.05, an 80% power, a total of 20 patients per group were needed.

Excitatory repetitive transcranial magnetic stimulation

Sample 1 received either 10 Hz rTMS (3000 pulses) or intermittent theta burst stimulation (iTBS; 600 pulses) at 120% of resting motor threshold, delivered with the PowerMAG EEG 100 Stimulator (MAG & More GmbH, München, Germany) using the butterfly coil PMD70-pCool. Sample 2 received 20 Hz rTMS (2000 pulses) at 110% of resting motor threshold, delivered with the DuoMAG XT-100 (DEYMED Diagnostic s.r.o., Hronov, Czech Republic) using the butterfly coil 70BF-Cool.

We did not use neuronavigation and, thus, do not have individual target coordinates available. To estimate the approximate distance between the stimulation targets corresponding to the three coil positions, we therefore extracted (average) target coordinates (in Montreal Neurological Institute (MNI) space) from previous publications: “6 cm rule”[8]: x=-28.8, y=35.1, z=46.2; Beam F3[9 10]: x=-40.6, y=41.7, z=34.3 and x=-43, y=46, z=32; Beam F3 Adjusted[11]: x=-38, y=44, z=26. The Euclidian distance between these targets is: “6 cm rule”–Beam $F3_{1/2}$: 18.0/22.8 mm; Beam $F3_{1/2}$–Beam F3 Adjusted: 9.0/8.1 mm.

Statistical analysis

Rating scale data followed a normal distribution according to the Kolmogorov-Smirnov test. Two-tailed t-tests for independent samples were used to compare averaged percent change of depression scores. Achieved power for paired t-tests were estimated by Cohen’s d_z. Sum scores as obtained through the course of treatment were subjected to two-factorial repeated measure ANOVAs with the two-level factor ‘localization method’ (“Beam F3”, “Beam F3 Adjusted” or “6 cm rule”) and the two-level factor ‘timepoint’ (baseline, after week 4/20 sessions of treatment). ANOVA effect sizes were estimated as partial η^2-values. Paired two-tailed t-tests were used to disentangle main effects. To test for homogeneity of response rates, the χ^2 independence test was used with an effect size as indicated by the φ coefficient.

Supplementary Table. Demographics and clinical characteristics of the intervention groups.

	Sample 1		Sample 2	
	**"Beam F3"**	**"Beam F3 Adjusted"**	**"Beam F3"**	**"6 cm rule"**
Number of patients	23	23	24	24
Age at start of treatment (years) [M ± SD]	52.7 ± 15.8	47.9 ± 17.2	52.3 ± 11.9	53.3 ± 11.4
Gender (women / men)	14 / 9	6 / 17	15 / 9	18 / 6
**Diagnosis**				
Major depression (number of patients)	21	22	23	23
Bipolar depression (number of patients)	2	1	1	1
**Psychiatric Medication**				
Antidepressants (number of patients)	22	19	19	23
Mood Stabilizers (number of patients)	4	2	1	2
Antipsychotics (number of patients)	15	9	13	15
Anxiolytics/Hypnotics (number of patients)	5	5	7	12
Benzodiazepines (number of patients)	7	4	5	9
**Baseline depression scores**				
BDI–II [M ± SD]	31.0 ± 10.0	32.0 ± 9.8		
HAMD–17 [M ± SD]	18.7 ± 5.5	17.4 ± 6.3		
BDI [M ± SD]			28.9 ± 11.0	31.3 ± 10.3

BDI, Beck Depression Inventory; BDI–II, Beck Depression Inventory revised; HAMD–17, Hamilton Depression Rating Scale; iTBS, intermittent theta burst stimulation; M, mean; Md, median; SD, standard deviation;

Supplementary Results

To exclude an impact of the excitatory stimulation protocol in sample 1 (10 Hz rTMS: n=21; iTBS n=25), we employed supplementary ANOVAs (for depression scores at each timepoint as well as change scores) and binary logistic regression analyses (for number of non-/responders). That no significant effects were found (all *p*>0.373) is in line with the results of a randomized non-inferiority trial indicating similar effectiveness.[12]

Given recent evidence that women (as compared to men) experience more favorable outcomes of accelerated rTMS[13], we used binary logistic and linear regression analyses to assess whether gender also represents a significant factor for response to regular (non-accelerated) rTMS in our study. Our supplementary analyses showed that gender did not predict therapeutic outcome in any of the employed samples (sample 1, sample 2, combined samples), neither non-/response (HAMD–17: *p*=0.730, BDI/-II all *p*>0.802) nor percent change in depression scores (HAMD–17: *p*=0.745, BDI/-II: all *p*>0.140).

To investigate whether the employed stimulation target localizations differentially influence dysphoric or anxiosomatic symptoms[14], we re-analyzed our data with focus on dysphoric and anxiosomatic symptom clusters, respectively. There was no difference between “Beam F3” and “Beam F3 Adjusted” in head-to-head comparisons of averaged percent change of dysphoric or anxiosomatic symptoms (HAMD–17: all *p*>0.339; BDI–II: all *p*>0.789). The 2 × 2 ANOVA indicated no interaction between ‘localization method’ and ‘timepoint’ for both dysphoric and anxiosomatic symptom clusters (HAMD–17: all p>0.521; BDI–II: all *p*>0.896). Response rates were equally distributed (to non-response rates) across the “Beam F3” and “Beam F3 Adjusted” localization methods for both dysphoric and anxiosomatic symptom clusters (HAMD–17: all *p*>0.907; BDI–II: all *p*>0.475).

There was also no difference between “Beam F3” and the “6 cm rule” in head-to-head comparisons of averaged percent change of dysphoric or anxiosomatic symptoms (BDI: all *p*>0.369). Also here, the 2 × 2 ANOVA indicated no interaction between ‘localization method’ and ‘timepoint’ for both dysphoric and anxiosomatic symptom clusters (BDI: all *p*>0.603). Also across the “Beam F3” and the “6 cm rule” localization methods, response rates for both dysphoric and anxiosomatic symptom clusters were equally distributed (BDI: all *p*>0.647).

Supplementary Discussion

Based on the employed sample sizes and standard deviations obtained from the comparisons in our own study, power analyses confirmed that our study design allowed for detecting changes of less than 32% change in Beck Depression Inventory scores, i.e., of less than the minimum clinically important difference (MCID) in participants with treatment-resistant depression[1].

Based on our results, we also calculated the number of participants needed to achieve 80% power for the primary outcomes of percent change in depression scales. In the “Beam F3” vs. “Beam F3 Adjusted” comparison, the computed sample sizes were n = 872,098 for the BDI-II and n = 1858 for the HAMD-17. In the Beam F3 vs. 6 cm rule comparison, the computed sample size was n = 1396 for the BDI.

Supplementary references

1. Button KS, Kounali D, Thomas L, et al. Minimal clinically important difference on the Beck Depression Inventory--II according to the patient's perspective. *Psychol Med* 2015;45(15):3269–79.
2. Bakker N, Shahab S, Giacobbe P, et al. rTMS of the dorsomedial prefrontal cortex for major depression: safety, tolerability, effectiveness, and outcome predictors for 10 Hz versus intermittent theta-burst stimulation. *Brain Stimul* 2015;8(2):208–15.
3. Hinchman CA, Fried PJ, Jannati A, et al. Corticomotor plasticity as a predictor of response to high frequency transcranial magnetic stimulation treatment for major depressive disorder. *J Affect Disord* 2022;303:114–22.
4. Kelly MS, Oliveira-Maia AJ, Bernstein M, et al. Initial response to transcranial magnetic stimulation treatment for depression predicts subsequent response. *J Neuropsychiatry Clin Neurosci* 2017;29(2):179–82.
5. Miron JP, Voetterl H, Fox L, et al. Optimized repetitive transcranial magnetic stimulation techniques for the treatment of major depression: A proof of concept study. *Psychiatry Res* 2021;298:113790.
6. Price GW, Lee JW, Garvey CA, et al. The use of background EEG activity to determine stimulus timing as a means of improving rTMS efficacy in the treatment of depression: a controlled comparison with standard techniques. *Brain Stimul* 2010;3(3):140–52.
7. Schulze L, Remington G, Giacobbe P, et al. Effect of antipsychotic pharmacotherapy on clinical outcomes of intermittent theta-burst stimulation for refractory depression. *J Psychopharmacol* 2017;31(3):312–19.
8. Bradfield NI, Reutens DC, Chen J, et al. Stereotaxic localisation of the dorsolateral prefrontal cortex for transcranial magnetic stimulation is superior to the standard reference position. *Aust N Z J Psychiatry* 2012;46(3):232–9.
9. Cash RFH, Zalesky A, Thomson RH, et al. Subgenual functional connectivity predicts antidepressant treatment response to transcranial magnetic stimulation: Independent validation and evaluation of personalization. *Biol Psychiatry* 2019;86(2):e5–e7.
10. Trapp NT, Bruss J, King Johnson M, et al. Reliability of targeting methods in TMS for depression: Beam F3 vs. 5.5 cm. *Brain Stimul* 2020;13(3):578–81.
11. Mir-Moghtadaei A, Caballero R, Fried P, et al. Concordance Between BeamF3 and MRI-neuronavigated target sites for repetitive transcranial magnetic stimulation of the left dorsolateral prefrontal cortex. *Brain Stimul* 2015;8(5):965–73.
12. Blumberger DM, Vila-Rodriguez F, Thorpe KE, et al. Effectiveness of theta burst versus high-frequency repetitive transcranial magnetic stimulation in patients with depression (THREE-D): A randomised non-inferiority trial. *Lancet* 2018;391(10131):1683–92.
13. DeSouza DD, Nakano E, Hoang V, et al. Real-world outcomes and predictors of accelerated rTMS treatment response for treatment-resistant depression. *medRxiv* 2024:2024.05.05.24306898. doi: 10.1101/2024.05.05.24306898
14. Siddiqi SH, Taylor SF, Cooke D, et al. Distinct symptom-specific treatment targets for circuit-based neuromodulation. *Am J Psychiatry* 2020;177(5):435–46.
